# Baseline Levels of Influenza-Specific CD4 Memory T-Cells Affect T-Cell Responses to Influenza Vaccines

**DOI:** 10.1371/journal.pone.0002574

**Published:** 2008-07-02

**Authors:** Xiao-Song He, Tyson H. Holmes, Sanae Sasaki, Maria C. Jaimes, George W. Kemble, Cornelia L. Dekker, Ann M. Arvin, Harry B. Greenberg

**Affiliations:** 1 Department of Medicine, Stanford University School of Medicine, Stanford, California, United States of America; 2 VA Palo Alto Health Care System, Palo Alto, California, United States of America; 3 Department of Health Research and Policy (Biostatistics), Stanford University School of Medicine, Stanford, California, United States of America; 4 MedImmune Vaccines, Mountain View, California, United States of America; 5 Department of Pediatrics, Stanford University School of Medicine, Stanford, California, United States of America; 6 Department of Microbiology and Immunology, Stanford University School of Medicine, Stanford, California, United States of America; Centre de Recherche Public-Santé, Luxembourg

## Abstract

**Background:**

Factors affecting immune responses to influenza vaccines have not been studied systematically. We hypothesized that T-cell and antibody responses to the vaccines are functions of pre-existing host immunity against influenza antigens.

**Methodology/Principal Findings:**

During the 2004 and 2005 influenza seasons, we have collected data on cellular and humoral immune reactivity to influenza virus in blood samples collected before and after immunization with inactivated or live attenuated influenza vaccines in healthy children and adults. We first used cross-validated lasso regression on the 2004 dataset to identify a group of candidate baseline correlates with T-cell and antibody responses to vaccines, defined as fold-increase in influenza-specific T-cells and serum HAI titer after vaccination. The following baseline parameters were examined: percentages of influenza-reactive IFN-γ^+^ cells in T and NK cell subsets, percentages of influenza-specific memory B-cells, HAI titer, age, and type of vaccine. The candidate baseline correlates were then tested with the independent 2005 dataset. Baseline percentage of influenza-specific IFN-γ^+^ CD4 T-cells was identified as a significant correlate of CD4 and CD8 T-cell responses, with lower baseline levels associated with larger T-cell responses. Baseline HAI titer and vaccine type were identified as significant correlates for HAI response, with lower baseline levels and the inactivated vaccine associated with larger HAI responses. Previously we reported that baseline levels of CD56^dim^ NK reactivity against influenza virus inversely correlated with the immediate T-cell response to vaccination, and that NK reactivity induced by influenza virus depended on IL-2 produced by influenza-specific memory T-cells. Taken together these results suggest a novel mechanism for the homeostasis of virus-specific T-cells, which involves interaction between memory helper T-cells, CD56^dim^ NK and DC.

**Significance:**

These results demonstrate that assessment of baseline biomarkers may predict immunologic outcome of influenza vaccination and may reveal some of the mechanisms responsible for variable immune responses following vaccination and natural infection.

## Introduction

Influenza viruses are major respiratory tract pathogens for people of all ages, especially the elderly and very young [1]. Currently two types of influenza vaccines are available: the inactivated trivalent influenza vaccine (TIV), given intramuscularly [2], and the live attenuated influenza vaccine (LAIV), administered intranasally [3]. TIV is approved for use in people ages 6 months or older, LAIV is approved for use in persons 2–49 years of age. Both vaccines are considered safe and effective for the designated age groups, although a recent study found that in healthy children aged 6 months–4 years, LAIV had significantly better efficacy than TIV for both antigenically well-matched and drifted strains [4]. In contrast, it was reported that in healthy adults aged 18–49, TIV and LAIV were similarly effective against drifted type A (H3N2) viruses, but that TIV was superior against type B infections [5].

Most adults and older children have pre-existing immunity against influenza viruses due to prior infection or vaccination [6], [7], [8]. However, antigenic drift of influenza virus, which is caused by accumulation of point mutations in viral genes encoding the two surface proteins, hemagglutinin and neuraminidase, occurs both in influenza A and B viruses [9], [10]. An individual who was infected by or vaccinated against previously circulated influenza viruses may be susceptible to a new virus strain. Therefore, the influenza vaccine is reformulated each year based on international surveillance that predicts which virus strains will circulate in the coming year.

Antibodies to hemagglutinin and neuraminidase have been associated with protection from disease and/or viral replication after natural influenza infection or vaccination in adults and children [11], [12], [13], [14], [15], [16], [17]. Based primarily on studies in animal models, T-cell responses are also thought to play an important role in clearing influenza virus infection [18], [19], [20], [21], [22], [23], [24]. Much less is known regarding the role of innate immune responses during influenza infection or following vaccination. Most of the previous immunological studies on vaccination focused on single or few adaptive immune parameters, such as the titer of virus-specific antibody or number of virus-specific T-cells. However, the complex interplay of factors affecting these immune responses, which are critical for understanding the efficacy of vaccination, have not been investigated systematically, especially for the cellular immune responses.

During the 2004 and 2005 influenza seasons, we carried out a comprehensive study to investigate humoral and cellular immune responses in children and adults immunized with either LAIV or TIV. The following parameters of the adaptive and innate immune compartments were measured with blood samples collected before and after vaccination: the percentage and phenotype of influenza-specific T-cells, the percentage of influenza-reactive IFN-γ–producing CD56^bright^ and CD56^dim^ NK cells, the percentage of influenza virus-specific IgG and IgA memory B-cells and antibody-secreting effector B-cells, and the titer of serum hemagglutination inhibition (HAI) antibodies. Some of these results have been reported in our previous publications that focused on the comparison of these responses between children and adults and between TIV and LAIV recipients [7], [8], [25].

An immune response is the outcome of orchestrated interactions between foreign antigen and host immune cells, initiated upon the entry of a pathogen or vaccine into the body. These interactions differ among people based in part on different levels of immune memory resulting from previous related infection or immunization. The immune memory, including virus-specific antibody and memory B-cells and T-cells, can be quantitatively measured with different assays at the time of vaccination. In addition, the immune responses may also be influenced by host factors such as age that affects function of immune cells, type of vaccine that affects pathways of antigen presentation, and host genetic characteristics. In the current study, we considered immune response to vaccination as a function of multiple demographic and immune variables, and explored the relationship between the baseline immune parameters and immune responses to vaccination, defined as changes of the parameters after vaccination. We focused on three of our measured parameters that are believed to represent critical characteristics of protective immunity: HAI titer and the frequency of influenza-specific CD4 and CD8 T-cells in the peripheral blood.

A distinct feature of this study is that analysis made effective use of two separate and independent datasets from the same population. The first dataset was collected during the 2004 influenza season. This sample was used to identify a list of candidate baseline correlates with each immune response. The second dataset was collected during the 2005 influenza season, in which a different influenza A/H3N2 strain was used in both the vaccines and the assays. This second dataset allowed us to assess which of the putative correlations were sufficiently robust to sampling error to remain statistically detectable in a new, independent sample.

## Methods

### Human participants and vaccination protocols

Prior to the fall 2004 and 2005 influenza seasons, children 5–9 years of age and adults 22–49 years of age were enrolled into a multi-project influenza vaccine study. The study protocol was approved by the institutional review board at Stanford University. Written informed consent was obtained from participants or their parents while assent was obtained from children 7 years and older. For the current analysis, we excluded all 2004 participants from the 2005 dataset, so that the 2004 and 2005 datasets had no participants in common and thus represent statistically independent samplings. Demographic information on the included study participants are summarized in Table 1. Participants were immunized with either TIV (Fluzone, Sanofi Pasteur) or LAIV (FluMist, MedImmune) following current guidelines for influenza vaccination. All the adult participants in the 2004 study were participants of a study in the previous fall 2003 influenza season, in which they were randomized in a 1∶1 ratio to receive either TIV or LAIV. For the 2004 study, these participants were immunized with the same type of vaccine as they received in the previous year. All adult participants in the 2005 study were randomized in a 1∶1 ratio to receive either TIV or LAIV. The 5–9 year old children were randomized in a 1∶1 ratio to receive either TIV or LAIV in both years. For those influenza vaccine-naïve children, a second dose of the same vaccine was given at approximately 28 days (for TIV) or 42 days (for LAIV) after the first dose according to recommendations. The 2004 TIV and LAIV vaccines both contained A/New Caledonia/20/99 (H1N1) and A/Wyoming/03/2003 (H3N2) strains. The third strain was B/Jiangsu/10/2003 in TIV and B/Jilin/20/2003 in LAIV. The 2005, TIV and LAIV vaccines both contained the A/New Caledonia/20/99 (H1N1) and B/Jiangsu/10/2003 (B) strains. The third strain was A/California/7/2004 (H3N2) in LAIV and A/NewYork/55/2004 (H3N2, an A/California/7/2004-like strain) in TIV.

**Table 1:** Demographic information of the study population.

Study year	Age group	Vaccine group	N (female/male^1^)	Age^1^ mean±SD
2004	Child 5–9 yr	LAIV	13 (6/7)	6.9±1.4
		TIV	15 (3/12)	7.1±1.0
	Adult 22–49 yr	LAIV	19 (11/8)	29.7±5.8
		TIV	18 (10/8)	32.6±8.2
2005	Child 5–9 yr	LAIV	8 (3/5)	6.3±1.0
		TIV	12 (5/7)	6.8±1.8
	Adult 22–49 yr	LAIV	20 (15/5)	31.5±8.0
		TIV	16 (13/3)	31.1±9.4

1 We did not detect a difference in gender composition (logistic regression p = 0.07) or in natural-logarithm transformed age (linear regression p = 0.42) between the 2004 and 2005 populations.

### Assays for immune parameters

Assay results from blood samples collected on day 0 (pre-vaccination) and day 28–42 (post-vaccination) were used in this analysis. The percentages of IFN-γ–producing CD4 and CD8 T-cells and CD56^bright^ and CD56^dim^ NK cells were measured with an IFN-γ flow cytometric assay after incubating PBMC with a live influenza A (fluA) H3N2 strain for 17 hours [7]. The percentage of influenza-specific memory IgA and IgG B-cells were measured with a two-color ELISPOT assay after culturing PBMC with polyclonal stimulation for 5 days [8]. The titer of serum HAI antibodies against fluA H3N2 was measured as previously described [8]. The PBMC samples collected during the 2004 or 2005 season were tested with either TIV (for ELISPOT), or the fluA H3N2 strain similar to the vaccine component of the same season (for IFN-γ flow cytometry and HAI assays), respectively. For the IFN-γ and HAI assays, we focused on results with H3N2 subtype virus because influenza A/H3N2 has, in recent years, generally been a more virulent strain compared to other strains and has exhibited more antigenic variation from season to season. In addition, the H3N2 component differed between the 2004 and 2005 vaccines, making any consistent finding between our two samples indicative of a more robust result.

### Identification of baseline correlates with immune responses with the 2004 dataset

The 2004 dataset was used to identify candidate baseline correlates of three immune responses specific for the fluA H3N2 strain: CD4 T-cell response, CD8 T-cell response, and HAI antibody response. These responses were defined as fold-change from baseline (day 0) in percentage of fluA-specific IFN-γ^+^ CD4 and CD8 T-cells and fold-change from baseline in HAI titer of the post-vaccination blood samples. One vaccine variable (TIV vs. LAIV), two host age variable (less than 10 years vs. at least 18 years, age in years) and the following 7 baseline immune variables were evaluated for their correlations with each of the three immune responses: percentage of IFN-γ^+^ CD4 T-cells, percentage of IFN-γ^+^ CD8 T-cells, percentage of IFN-γ^+^ CD56^bright^ NK cells, percentage of IFN-γ^+^ CD56^dim^ NK cells, percentage of influenza-specific IgA memory B-cells, percentage of influenza-specific IgG memory B-cells, and HAI titer. The percentage of IFN-γ^+^ cells was logarithm-base-10 transformed, while HAI titers were logarithm-base-2 transformed, for all analyses. Vaccine type and age group were coded as indicator variables [26] (pp. 455–457) for analysis. We chose to include both age in years and age group as possible baseline correlates because, a priori, we did not know to what extent variation in ages within adults and within children may contribute to prediction beyond that by age groups alone (see results).

In the 2004 sample, we employed lasso regression [27] with five-fold cross-validation [28](p. 216) for the specific purpose of empirically identifying a list of candidate baseline correlates with immune response (and not for obtaining estimates of regression coefficients). Lasso regression generates a sequence (path) along which regression variables are removed one by one. For five-fold cross-validation we randomly split the 2004 data set into five parts of approximately equal quantities of participants. A lasso regression model was fit to the data on the last four parts combined (training) and then the data from the first part were applied to that fitted model and error was measured via the predicted residual sum of squares (PRESS) statistic (validation). The process was then repeated four more times, allowing each part (2, 3, 4, 5) to serve separately as the validation data for a training on the other four parts combined. When the sample size is modest, such as in this dataset of 71 participants, the cross-validation component is small (71/5≈14 participants), making cross-validation results less stable. To enhance stability, we repeated cross-validation 150 times, each time using a separate random splitting of the 2004 data set into five parts of approximately equal quantities of participants. This yielded 150 removal sequences of candidate baseline correlates. We identified as most robust that specific removal sequence which occurred most frequently among these 150. From this specific sequence, the optimal subset retained for independent testing with the 2005 data was that combination of candidate baseline correlates which had the smallest cross-validated PRESS statistic. Within this optimal subset, we defined strength of candidacy according to order of removal had variable selection been allowed to proceed below the optimum. This allowed us to address any co-linearity among candidates by prioritizing order of hypothesis testing from strongest to weakest candidates. The entire variable-selection analysis was performed separately for each of the three immune responses.

### Validation of baseline correlates for immune responses with the 2005 dataset

Using the independent 2005 sample, we performed ordinary multiple regression of each immune response on its subset of candidate baseline correlates identified from 2004. Using Type I sums of squares [29], each candidate baseline correlate was tested for association with immune response after having adjusted for all other baseline correlates of stronger candidacy in the subset. An association was declared statistically significant for attained significance levels of *p*<0.05.

## Results

To identify candidate baseline correlates for CD4 T-cell, CD8 T-cell and antibody responses to vaccination, we used the 2004 dataset with 71 participants. For a few participants, data were missing on either baseline (day 0) or day 28: 3 missing observations for baseline IgA memory B-cells, one for baseline HAI titer, and one each for day 28 HAI titer, CD4 and CD8 T-cells. Lasso regression analysis of this dataset identified one candidate baseline correlate for fold-change in percentage of fluA-specific CD4 T-cells, which was the baseline percentage of fluA-specific CD4 T-cells. Two candidate baseline correlates were identified for fold-change in percentage of flu-specific CD8 T-cells. These were adult age group (weakest candidate) and baseline percentage of flu-specific CD4 T-cells (strongest candidate). Three candidate baseline correlates were identified for fold-change in HAI titer. In order of increasing strength of candidacy these were vaccine type, age in years and baseline HAI titer.

**Figure 1:**
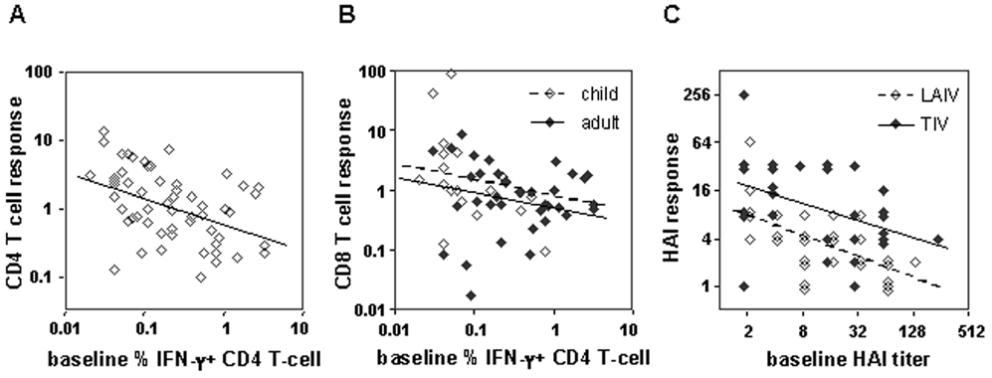
Scatter plot of immune responses versus identified baseline correlates for the 2005 sample. Immune responses are defined as the post-vaccination fold-change of each immune parameter from its baseline level. Each open or closed circle on the plot is an observation from a single participant. Lines indicate fit of the multiple regression model to the data. A. CD4 T-cell response versus baseline percentage of fluA-specific IFN-γ^+^ CD4 T-cells. B. CD8 T-cell response versus baseline percentage of fluA-specific IFN-γ^+^ CD4 T-cells. C. HAI response versus baseline HAI titer.

Next we used regression analysis to test each of the candidate baseline correlates, using the independent 2005 dataset that includes a total of 56 adult and child participants. HAI data were missing for one child participant. Results are provided in Table 2. Baseline percentage of fluA-specific CD4 was associated with fold-change in percentage of flu-specific CD4 T-cells (*p* = 0.0003). The negative estimate of the regression coefficient indicates that the relationship between this baseline correlate and immune response is inverse. Namely, post-vaccination fold-change from baseline declined with increasing percentage at baseline (Fig. 1A). Fold-change in percentage of fluA-specific CD8 T-cells was associated with baseline percentage of fluA-specific CD4 T-cells (*p* = 0.0397) but not with age group in the 2005 data (*p* = 0.3684, Fig. 1B). Fold-change in HAI was associated with baseline HAI titer (*p*<0.0001) and with vaccine type (*p* = 0.0098) but not with age in years (*p* = 0.5864). The estimated regression coefficient for vaccine type (LAIV) is negative, indicating that fold-change from baseline in HAI titer is smaller after LAIV than after TIV vaccination (Fig. 1C).

**Table 2:** Estimated regression coefficients from the 2005 sample.

Immune response	Candidate Baseline Correlate	Estimated Regression Coefficient	*p* value	Partial Correlation Coefficient *r_p_*	Estimated Coefficient of Multiple Correlation *R*	Estimated Coefficient of Multiple Determination *R* ^2^
CD4 T-cell response	% CD4	-0.41	0.0003	−0.47	0.47	0.22
CD8 T-cell response	% CD4	-0.23	0.0397	−0.28	0.3	0.09
	Adult Group	−0.18	0.3684	−0.12		
HAI response	Log_2_ HAI titer	−0.43	0.0002	−0.47	0.62	0.39
	Age (years)	−0.009	0.5668	−0.13		
	LAIV Vaccine Type	−1.39	0.0007	−0.45		

Within each immune response, candidate baseline correlates are ordered from strongest (top row) to weakest (bottom row) candidacy based on the 2004 lasso regression. Reported *p*-values are for the null hypothesis that the regression coefficient is equal to zero. Partial correlation coefficients are simple correlations [31](p. 426) for the first row of each immune response, because these are not adjusted for any other candidate correlates.

Six separate hypothesis tests were performed on the 2005 sample, across which the probability of a Type I error compounds. If we take a conservative approach and adjust for multiple testing [30], three of the associations between candidate baseline correlates and immune responses remain statistically significant: baseline percentage of fluA-specific CD4 T-cells with fold-change in percentage of fluA-specific CD4 T-cells, baseline HAI titer with fold-change in HAI and vaccine type with fold-change in HAI titer. Of note, these three associations remain statistically detectable after two checks on their robustness-testing in a new independent sample and correction for multiple testing.

Beyond identifying where association may exist, we can quantify strength of association with estimates of correlation parameters. Table 2 also provides estimates of the strength of association between each individual candidate baseline correlate and its immune response using estimates of Pearson partial correlation *r_p_* coefficients [31](pp. 426–428). Each partial correlation coefficient is adjusted for all stronger candidate baseline correlates with that immune response (Table 2). For example, the association between fold-change in HAI titer and vaccine type is adjusted for baseline HAI titer and age in years. The parameter *r_p_* can range in value from −1 to 1, with 0 representing no linear correlation. Estimated partial correlations for our data range from small (−0.12) to modest (−0.49).

We can also estimate the strength of association between each immune response and its corresponding full set of candidate baseline correlates identified from the 2004 sample (one correlate for CD4 T-cell, two for CD8 T-cell and three for HAI responses) using estimates of the coefficient of multiple correlation *R* [26](p. 231). Theoretically, *R* values range from 0 for no association to 1 for perfect association. We observed *R* values of 0.3 to 0.57 (Table 2). The square of *R* is the coefficient of multiple determination. *R* ^2^ can be interpreted as the proportion of the variance in the immune response explained by its candidate baseline correlates [26](pp. 230–231). These values are 0.09 for CD8 T-cell response, 0.22 for CD4 T-cell response, and 0.33 for HAI response for the full set of correlates identified in the 2004 sample. That is, up to 9% to 33% of the immune responses can be explained by the combined baseline correlates that we identified from 2004. If only those variables of statistically significant association with immune response are included (as assessed with the 2005 data), these multiple correlation and determination estimates would be lower.

## Discussion

Our working hypothesis for this study is that immune responses to vaccine antigens are a function of overall immunity against the antigen at the time of vaccination, as well as a function of the host and vaccine variables that influence activity of immune cells. To test this hypothesis, we searched among a total of seven influenza-specific baseline immune variables related to either the adaptive or innate immune system, two age-related host variables and one vaccine variable for baseline correlates of T-cell and antibody responses to influenza vaccination. We identified baseline levels of fluA-specific memory CD4 T-cells as a significant negative baseline correlate with CD4 and CD8 T-cell responses, and baseline levels of HAI titer and type of vaccine (LAIV) as significant negative correlates with antibody response.

Among the immune variables we examined, CD4 helper T-cells are required for B-cell responses, which are related to antibody responses, and for cytotoxic CD8 T-cell responses. Pre-existing memory B-cells can be activated and develop into high affinity antibody-secreting effector B-cells upon encountering related antigens. In addition, activated B-cells may secrete cytokines and serve as antigen-presenting cells (APC) for T-cell responses [32]. The relationship between the levels of pre-existing memory T-cells and B-cells in the periphery and the subsequent B- and T-cell responses to a new influenza infection or immunization is not known. NK cells, as a member of the innate immune system, provide a first line of defense against viral infection, which may affect the subsequent adaptive T-cell responses [33], [34], [35]. However, most previous immunological studies of influenza vaccines have focused on adaptive immunity, i.e. antibody and T-cell responses, and rarely on innate immunity. In limited influenza vaccine studies, an enhanced NK cytotoxicity has been seen following vaccination in some studies [36], [37] but not in others [38].

In addition to the immune variables, we chose age in years and age group (child vs. adult) as host factors, and type of vaccine as another factor to be considered, based on our previous findings on the different cellular immune responses to influenza vaccination between the different age and vaccine groups [7], [8], [25], [39]. LAIV and TIV are administered through different routes and elicit CD8 T-cell responses through different antigen presentation pathways. LAIV is administered to the nasal mucosal surface. Similar to natural infection with wild-type virus, LAIV is presumed to undergo replication at the site of immunization and infects APC. This viral replication may be limited to variable degrees based on the strength of innate and adaptive immunity, which are likely to affect the magnitude of subsequent adaptive immune response to LAIV. In contrast, TIV is administered by intramuscular injection and induces systemic immune responses. Hence the variable microenviroment in T-cell activation sites and different pathways of antigen presentation associated with these two vaccines may result in different immune responses to the two vaccines.

The 2004 and 2005 datasets have no participants in common and thus represent statistically independent samplings. This allowed us to use one dataset to identify candidate baseline correlates with immune response and the other to independently verify which of these candidate baseline correlates are statistically associated with immune response. Testing for association in an independent dataset is statistically advantageous. Use of an independent sample strengthens findings by identifying associations that are less affected by the idiosyncrasies of the sample used for initially selecting candidate baseline correlates. In particular, bias is avoided in estimates of p-values and regression coefficients [40] that arise when conducting hypothesis tests on those same data that suggested which hypotheses to test (“data snooping,” [26] pp. 724–725).

Based solely on serological data, previous studies in our group and others have shown that lower baseline levels of influenza-specific antibody and vaccination with TIV rather than LAIV were associated with greater antibody response after vaccination [8], [41], [42]. These same factors have emerged out of the 10 variables that we examined as the only baseline correlates with antibody response to influenza vaccination, providing further evidence of the robustness of the findings reported here.

Our analysis also identified baseline levels of fluA-specific IFN-γ^+^ CD4 T-cells as a significant baseline correlate for both CD4 and CD8 T-cell responses to influenza vaccination with an inverse correlation, even though the association is only modest in strength. What is the possible underlying mechanism for this association? Most adults and older children have been exposed to previous influenza infection or vaccination [6], [7], [8], and therefore have certain levels of influenza-specific memory T-cells in peripheral blood [7]. Previously we reported that when PBMC were incubated with fluA, the IFN-γ response of NK cells depended on the T-cell population, and the effects of T-cells could be replaced by recombinant IL-2 [43]. IL-2 is a cytokine transiently produced by activated T-cells, especially CD4 helper T-cells [44]. These findings indicate that IL-2 produced by fluA-specific memory CD4 T-cells is involved in the response of NK cells to fluA.

DC play a central role in both innate and adaptive immunity. DC process and present viral antigens to specific T-cells, resulting in activation and amplification of virus-specific T-cells, which constitute the primary or secondary T-cell responses to viral infection or vaccination. Depending on their maturity and functional status, DC can also tolerize T-cells, rendering them anergic to cognate antigens [45]. Studies using in vitro cultured NK cells and DC have shown that NK-DC interaction may result in their reciprocal activation, as well as inhibition of DC by NK, in different circumstances [34], [35], [46], [47], [48]. At low NK/DC ratios DC responses were amplified dramatically, while at high NK/DC ratios DC responses were inhibited completely. The inhibition of DC functions by NK cells was mediated by the potent DC killing activity of the autologous NKs cells [47], which is most likely mediated by the CD56^dim^ NK cell subset that expresses high levels of perforin [49]. Therefore, depending on the magnitude of CD56^dim^ NK activity during the early stage of infection, the NK cells could either enhance or suppress subsequent adaptive T-cell responses by activating or inhibiting DC. Of note, CD56^dim^ NK cells stimulated by tumor cells were both cytolytic and IFNγ-producing [50], suggesting an association between these two effector functions of the CD56^dim^ NK population. Of special interest, previously we showed that the immediate T-cell responses after vaccination, defined as the fold-change in percentage of IFN-γ^+^ CD4 and CD8 T-cells by day 10 after vaccination, was inversely correlated with baseline levels of fluA-reactive IFN-γ^+^ T-cells and NK cells and had the most direct association with percentage of IFN-γ^+^ cells in the CD56^dim^ NK subset [7].

**Figure 2:**
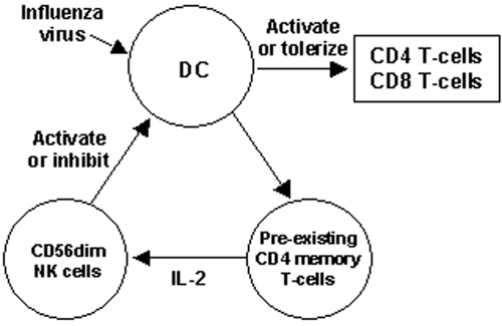
A model for the inverse association between T-cell responses to influenza antigen and the levels of pre-existing influenza-specific memory CD4 T-cells. Upon exposure to influenza virus or vaccine, pre-existing virus-specific memory CD4 T-cells secrete IL-2, which activates CD56^dim^ NK cells in a dose-dependent way. Depending on the activity of CD56^dim^ NK cells, DC are either activated or inhibited, which in turn modulate the subsequent T-cell responses to influenza virus accordingly.

Based on these observations, we propose the following model for the homeostasis of influenza-specific T-cells during repeated exposure to influenza infection and vaccination (Figure 2). When influenza virus or vaccine enters an individual with low levels of virus-specific memory CD4 T-cells, low levels of baseline reactivity of CD56^dim^ NK cells, due to low levels of IL-2 produced by the CD4 T-cells, favor the antigen-presenting functions of DC and lead to vigorous virus-specific T-cell responses. In contrast, high baseline levels of virus-specific memory CD4 T-cells should enhance NK cell reactivity upon re-exposure to the virus or vaccine antigens and inhibit DC function by CD56^dim^ NK cell-mediated killing of DC. This would lead to limited activation and expansion of virus-specific T-cells. Alternately or concurrently, the high levels of NK activity could reverse the function of DC from activation to tolerization of virus-specific T-cells and render them anergic, decreasing the production of IFN-γ by the virus-specific T-cell population.

Two of the candidate baseline correlates identified with the 2004 dataset were no longer significant when tested with the 2005 dataset. These were age group for CD8 T-cell response and age in years for HAI response. This could be explained by some differences in the samples for these two years. All the adult participants, but not the children, in the 2004 study received the same type of influenza vaccine in 2003 and 2004 flu seasons; while all adult and child participants in the 2005 study were randomized to received either LAIV or TIV. We found both baseline immune parameters as well as immune responses could be affected by immunization status in the prior year ([8] and data not shown). In addition, the IFN-γ flow cytometric assay in these two years used different fluA H3N2 strains matched to the vaccine antigen of each year. These two strains appear to induce IFN-γ production in T-cells and NK cells at different efficiencies (He et al., unpublished results).

Finally, we recognize that strengths of association are modest at best between identified baseline correlates and immune responses identified in the current study. However, it is encouraging that significant baseline correlates can be identified from our very limited set of baseline immune parameters after the stringent screening and testing presented here. In the current study, four out of the seven immune parameters examined with the 2004 sample pertain to IFN-γ production, which may not be the best parameter, and definitely not the only parameter, for characterizing the highly diversified functions of lymphocytes. TCR-based assays for antigen-specific T-cells, such as tetramer staining, may provide more accurate quantitative measurement for specific T-cells. Peptide-MHC microarry-based technology offers the ability to characterize and analyze multiple epitope-specific T-cell populations during immune responses [51]. Similarly, multicolor flow cytometry allows simultaneous measurement of multiple phosphorylated cellular signal molecules [52] as well as multiple cytokine production [53] in multiple immune cell subsets, providing a more complete quantitative and qualitative assessment for the quality of T-cell response that correlate with the immune response of disease in some models [53]. Together with development of other high-throughput analysis technologies, information on the function of immune systems, including many biomarkers that were not assessed in the current study but may be critical for immune responses, can be collected at unprecedented scale. As for statistical methods, variable-selection technologies are continuing to evolve, in particular with regard to their “oracle” properties, which is their ability to identify true correlates with immune response. Identification of baseline immune correlates with immune response will become increasingly reliable as nascent statistical and bioinformatical tools for data analysis are more thoroughly tested and become more widely available. This will not only lead to precise prediction of immune response of infection, vaccination and treatment, but also reveal the critical underlying mechanisms for the processes of disease and health.
